# Is impaired response inhibition independent of symptom dimensions in obsessive-compulsive disorder? Evidence from ERPs

**DOI:** 10.1038/srep10413

**Published:** 2015-05-20

**Authors:** Hui Lei, Xiongzhao Zhu, Jie Fan, Jiaojiao Dong, Cheng Zhou, Xiaocui Zhang, Mingtian Zhong

**Affiliations:** 1 Medical Psychological Institute, Second Xiangya Hospital, Central South University, Changsha 410011, P.R. China; 2 National Technology Institute of Psychiatry, Key Laboratory of Psychiatry and Mental Health of Hunan Province, Central South University, Changsha 410011, P.R. China; 3 Center for Studies of Psychological Application, South China Normal University, Guangzhou 510631, P.R. China

## Abstract

Impaired response inhibition has been consistently reported in patients diagnosed with obsessive-compulsive disorder (OCD). This clinically heterogeneous disorder is characterized by several symptom dimensions that may have distinct, but partially overlapping, neural correlates. The present study examined whether alterations in response inhibition may be related to symptom severity and symptom dimensions. Event-related potentials (ERPs) were recorded in a group of 42 medication-free OCD patients as well as 42 healthy controls during a stop-signal task. Symptom dimension scores were obtained using the Yale-Brown Obsessive Compulsive Scale symptom checklist. OCD patients showed longer stop-signal reaction times (SSRT, *p* < 0.01) and larger stop-N2 amplitudes (*p* < 0.01) compared to healthy controls. Neither the longer SSRT nor the larger stop-N2 scores were significantly correlated with symptom severity or present or lifetime OCD symptoms in OCD patients. These results indicate that deficient response inhibition is a common occurrence in OCD patients that is independent of global symptom severity and symptom dimensions. These data support the notion that impaired response inhibition may be a general attribute of patients with OCD.

Obsessive-compulsive disorder (OCD) is a clinically heterogeneous disorder characterized by the presence of obsessions (repetitive intrusive thoughts) and/or compulsions (repetitive ritualistic behaviors). Different clinical symptom dimensions present in patients are linked to differences in treatment response rates^1^, comorbidity patterns^2^, and genetic underpinnings^3^,^4^. Neuroimaging studies have found that clinical symptoms of OCD, which include as checking, symmetry, washing, and hoarding, likely reflect the activation of different brain regions and networks^5^,^6^,^7^,^8^. These findings indicate that the apparent phenotypic heterogeneity of OCD symptoms may reflect partially overlapping, yet distinct, disease mechanisms.

The term “symptom dimensions” for OCD patients refers to the thematic content of an individual’s obsessions and related compulsions^9^. The most common approach towards deriving clinical symptom dimensions for OCD is the factor analytic approach, which uses the Yale-Brown Obsessive Compulsive Scale-Symptom Checklist (Y-BOCS-SC)^10^. In the current literature, studies assessing OCD symptom dimensions have been inconsistent due to the use of different methodologies and varying sample sizes^9^. A recent item-level factor analysis of 1,224 OCD patients derived a five-factor model that contained the following factors: taboo, contamination/cleaning, doubts, superstitions/rituals, and symmetry/hoarding^3^. Notwithstanding the heterogeneity in clinical presentation, patients with OCD appear to have one common feature, namely, difficulty in suppressing repetitive and unpleasant thoughts and actions. This deficit of inhibitory control has long been theorized to be a central feature of OCD^11^.

Response inhibition is the process by which a motor action is withheld upon the appearance of a stop signal. Previous studies have shown impaired response inhibition in patients with OCD^12^,^13^,^14^,^15^. A recent meta-analysis reported that deficits in response inhibition may more prominent in OCD compared to related disorders such as Tourette’s syndrome or anxiety and mood disorders^16^. To systematically assess the degree to which response inhibition is affected in OCD patients, the current study aims to quantify response inhibition in patients with OCD and to assess to what degree this deficit may be related to symptom severity and different symptom dimensions.

The stop-signal task (SST) is a highly suitable and widely used paradigm for the study of response inhibition in clinical populations^17^. The duration of time needed for the individual to process the stop response (stop-signal reaction time, SSRT), or stop process latency, has been shown to be an important measure of the cognitive control processes that involved in halting an action. Previous studies have consistently observed poorer inhibitory performance in individuals with OCD relative to controls, reflected by longer SSRTs^12^,^13^,^14^,^15^,^18^. The impaired inhibition of simple motor responses was proposed to be an endophenotype for OCD, a notion that has been supported by studies describing impaired inhibition in unaffected first-degree relatives of individuals with OCD^13^,^14^,^18^.

Event-related potentials (ERPs) are small voltage fluctuations that can be recorded noninvasively from the intact human scalp. Measuring these signals allows researchers to directly investigate the temporal properties of neural activity that underlies inhibitory processing. During the SST task, the stop-signal N2 and stop-signal P3 components of the ERP are considered to represent response inhibition. The stop-signal N2 component, which peaks at approximately 200-250 ms, has been specifically associated with an early mechanism of inhibitory control^19^,^20^. It has been proposed that the N2 represents a “red flag” signal that is generated in the prefrontal cortex in order to trigger an inhibition of the motor response^19^. In contrast, the stop-signal P3, which peaks at 300-350 ms over central scalp regions in SST trials, reflects late stage monitoring of the inhibitory process outcome^20^.

Only one ERP study has been undertaken to characterize response inhibition using SST in patients with OCD^21^. In this earlier study, a relatively small sample size (n = 10) was selected for each group, and the stop-signal delay was randomly chosen with a rectangular distribution. The results showed that OCD patients have larger N2 amplitudes elicited by “stop” stimuli compared to controls. In the current study, we recruited a relatively large sample of OCD patients (n = 42). In addition, the stop-signal delay was varied using a tracking method that increased or decreased the stop-signal delay according to whether the response had been inhibited or not on the previous stop-signal trial^22^. The tracking method takes into account subject strategies of speed and variability and allows the overall inhibition probability to be maintained near 50%, which is useful in ERP research, as it results in roughly equal numbers of successful and failed inhibition trails. Despite growing evidence for altered response inhibition in OCD patients, the relationship between response inhibition and symptom severity/symptom dimensions has not been examined thoroughly.

Accordingly, the current study uses behavioral and electroencephalographic (EEG) measures of response inhibition in patients diagnosed with OCD to examine the relationship between these measures of response inhibition, symptom severity, and symptom dimension scores. Given the exploratory nature of the examination, we chose an unbiased approach without the formulation of an *a priori* hypothesis regarding relationship between the measurements of response inhibition and the symptoms severity and symptom dimension scores.

## Results

### Demographic and clinical characteristics

Demographical, clinical, and behavioral measures are presented in Table 1. The groups did not differ in mean age, years of education, or gender ratio. Patients with OCD scored higher on self-reported symptom severity (OCI-R: *t*(82) = 5.84, *p* < 0.001, *Cohen’s d* = 1.27; BDI-II: *t*(82) = 8.23, *p* < 0.001, *Cohen’s d* = 1.80). All patients endorsed more than one symptom dimension on the Y-BOCS Symptoms Checklist (Table 2).

### Task performance

The groups did not differ in median reaction time (RT) and accuracy on the go task (see Table 1). There was no difference between groups in the probability of successful inhibition. A significant difference in SSRT between control and OCD patient groups was observed, with longer SSRTs measured in OCD patients (*t*(82) = 2.94, *p* < 0.01, *Cohen’s d* = 0.64). In addition, a significant positive correlation was found between go RT and probability of successful inhibition (*r*(84) = 0.37, *p* < 0.01), whereas go RT was not associated with success rate in go trials or with SSRT. SSRT was also not significantly correlated with probability of successful inhibition.

### ERPs to the visual stop-signal

**Figure 1:**
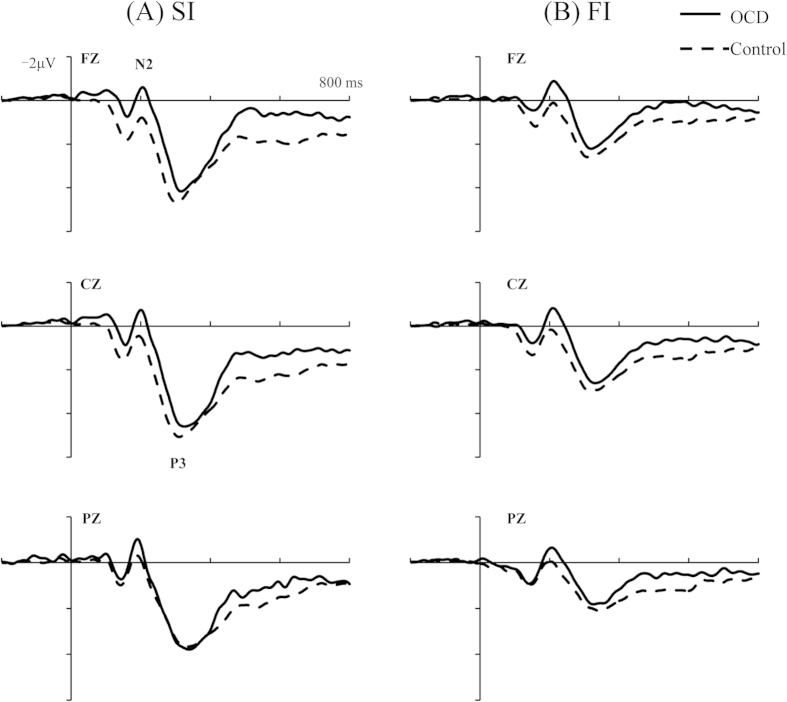
Grand average ERP waveforms time-locked to the stop-signal for Successful Inhibition (SI) and Failed Inhibition (FI) at 3 midline sites (Fz, Cz, Pz) for the OCD Group (solid line) and the Control Group (dashed line).

The grand average ERP amplitudes in response to the visual stop-signal in Successful Inhibition (SI) and Failed Inhibition (FI) trials for each group separately were presented in Fig. 1.

Amplitudes of the N2 component in response to the stop signal (Stop-N2) were most prominent at the Fz electrode site (*F*(2,164) = 3.39, *p* < 0.05, *η*^*2*^ = 0.04). Larger (more negative) N2 amplitudes were observed in the FI compared to the SI condition (*F*(1,82) = 8.87, *p* < 0.01, *η*^*2*^ = 0.10). A significant main effect of group was observed (*F*(1,82) = 9.84, *p* < 0.01, *η*^*2*^ = 0.11) indicating that patients with OCD had larger (more negative) N2 amplitudes compared to healthy control participants.

The Stop-N2 component showed longer latencies in the FI than in the SI condition (*F*(1,82) = 6.37, *p* < 0.05, *η*^*2*^ = 0.07), and larger amplitudes at the Fz than at the Cz or Pz electrode sites (*F*(2,164) = 11.17, *p* < 0.001, *η*^*2*^ = 0.12). The Stop-N^*2*^ latency did not differ between the OCD and control groups.

The ERP P3 component elicited in response to the stop signal (Stop-P3) was most prominent at the Cz electrode site (leads: *F*(2,164) = 39.37, *p* < 0.001, *η*^2^ = 0.32). A larger P3 amplitude was found in the SI compared to the FI condition (type: *F*(2,164) = 67.94, *p* < 0.001, *η*^2^ = 0.45). Stop-P3 amplitudes did not differ between the groups. The latencies were longer at the Cz and Pz leads than at the Fz lead (*F*(2,164) = 23.02, *p* < 0.001, *η*^*2*^ = 0.22). The latency did not differ between OCD and controls groups.

### Correlations with demographical and clinical variables

For the whole sample, there was a positive association between age and the amplitude of Stop-N2 in the SI and FI conditions (SI: *r* = 0.32, *p* < 0.01; FI: *r* = 0.33, *p* < 0.01), as well as the amplitude of Stop-P3 in the SI condition (*r* = 0.29, *p* < 0.01). Age was not associated with the SSRT or with the latency of the N2/P3 components. In the OCD patient sample, neither SSRT nor amplitude or latency of the N2/P3 elicited in response to the stop signal was significantly correlated with YBOCS, OCI-R, or BDI-II scores.

### Symptom dimension analyses

The behavioral (SSRT) and ERP (Stop-N2 amplitude in the SI or FI condition) indexes of response inhibition were entered as dependent variables separately in the multiple regression analyses. There were no significant relationships between each of the symptom dimension scores and the amplitude of Stop-N2 in the SI or FI condition. Likewise, there were no significant relationships between symptom dimension scores and the SSRT (Table 3). The data in Table 3 demonstrate that symptom dimension scores did not have significant, independent contributions to the amplitude of Stop-N2 or to the SSRT.

## Discussion

The current study took advantage of a large sample of medication-free patients to address the question of whether deficits in response inhibition in OCD patients are associated with the expression of specific symptoms. We found that the impaired response inhibition found in patients was independent of the severity of their OCD, as well as depressive, symptoms. We show that patients with OCD display longer latencies of the stop process (SSRT) compared to healthy controls. This finding is in agreement with other studies using the stop-signal paradigm^12^,^13^,^14^,^15^,^18^. The findings of consistently longer SSRT seem to support the notion that OCD patients have a longer time to stop the motor responses as the response inhibition deficit in these patients. Alternative interpretation of this finding is associated with the deficits in attention allocated to the motor control tasks in OCD patients, i.e. they usually allocate more attention sources to non-target events and as a consequence cannot shift their attention away from them, hence showing more rigidity in the motor system^23^,^24^. This may lead to more rigid “go” response associated with longer SSRT. No group differences were found in primary task performance. The fairly long go RTs in both groups may be associated with adjusting response strategies. Previous studies indicate that subjects trade speed in the go task for success as expecting stop signal to occur, or after unsuccessful inhibition or successful stopping^25^,^26^,^27^. We found a significant positive correlation between go RT and probability of successful inhibition, which also supports that the long go RTs might be attributed to the behavior adjustment for success. These findings indicate that the impaired inhibition in the OCD patients may not be attributable to a general slowing of cognitive processes but, rather, are at least partly due to specific deficits in the processing of the stop stimulus.

In the present study, the amplitude of the ERP N2 component elicited in response to the stop-signal, the Stop-N2, displayed a greater frontal maximum for failed than for successful inhibition trials, in agreement with previous studies^28^,^29^,^30^. One explanation for this result is that participants are already aware, at the time of the stop-signal presentation, that the inhibition will be ineffective on unsuccessful trials, thereby increasing the subjective importance of the stimulus and, in consequence, the amplitude of the Stop-N2^31^. Another interpretation could be that the Stop-N2 component reflects an evaluative process that detects the occurrence of conflict between the go and stop responses^20^. The conflict would thus be smaller on successful stop trials because the inhibitory response overrides the go response before it is executed. However, the Stop-N2 signal on failed trials is composed of a Stop-N2 response plus an error-related negativity elicited by the realization of an impending mistake. In line with a previous study, patients with OCD had larger Stop-N2 amplitudes across both successful and failed stop-signal trials. This result suggests that OCD responders required greater phasic inhibitory activation to inhibit a response. Or, the conflict between the go and inhibitory responses on stop trials could be larger in OCD patients than in healthy controls.

In the present study, the Stop-P3 component showed a greater central maximum for successful than for failed stop trails. This finding supports the concept that the P3 signal is related to inhibitory processing and is affected by the success of inhibition^28^,^29^. There were no Stop-P3 group effects in this study for either amplitude or latency. If the Stop-P3 component is associated with the subject’s success or failure to inhibit a response, then there should be no amplitude differences between the healthy and OCD groups, because the inhibition rates of two groups did not significantly differ. Therefore, our results further support the concept that the Stop-P3 component may be associated with the monitoring of the inhibitory process outcome^28^. No group differences in the amplitude of the Stop-P3 component were found; this, combined with a significant group difference in the amplitude of the Stop-N2, suggests that deficits in response inhibition manifested in the altered amplitudes observed in the Stop-N2 component.

The dimensional approach that we used is assumed to promote the identification of general and specific etiological factors that contribute to the development of OCD symptoms. Importantly, the current study shows that neither longer SSRT nor larger Stop-N2 amplitude is significantly correlated with OCD symptom dimensions for present or lifetime symptoms. This result might suggest that impaired response inhibition represents a neural correlate generally related to OCD, and is thus a general attribute of patients with OCD independent of the presence of symptoms on certain dimensions. This is in accordance with studies indicating a substantial overlap in gene expression, environmental influences, neurobiology, and clinical appearance across OCD symptom dimensions^3^,^4^,^8^, supporting the utility of a general OCD diagnosis.

Response inhibition has been associated with the activation of the inferior frontal gyrus (IFG), anterior insula, anterior cingulated cortex (ACC), dorsolateral prefrontal cortex (DLPFC), pre-supplementary motor area (pre-SMA), and parietal regions^32^,^33^. Additionally, structural and functional neural correlates of impaired motor response inhibition in OCD patients have been identified^14^,^18^,^34^,^35^,^36^. For example, deficits in response inhibition in OCD patients were associated with increased gray matter volume in the ACC, putamen, caudate, amygdala, parietal areas, and the cerebellum, and decreased gray matter volume in the orbitofrontal cortex, IFG, ACC, premotor cortex, and regions in the temporal cortex^18^. Functional MRI studies have also correlated defective response inhibition with the decreased activation of the IFG and inferior parietal cortex and increased activation of the left pre-SMA in OCD patients^14^. Although several neuroimaging studies led to a relative consensus that OCD is an etiologically heterogeneous disorder with both overlapping and distinct neural correlates across symptom dimensions^5^,^6^,^7^,^8^, future neuroimaging studies will be needed to further determine whether different symptom dimensions in OCD share the neural substrates that underlie deficits in response inhibition.

It is worth noting that impaired response inhibition is not specific to patients with OCD; previous studies have reported response inhibition deficits in other psychopathological and neurological disorders. Some disorders, such as schizophrenia^37^,^38^ and several neurodevelopmental disorders (e.g. autism^39^, attention-deficit/ hyperactivity disorder (ADHD)^40^, Tourette’s syndrome^41^) are associated with response inhibitory deficits which is a characteristic of these disorders. Beside these disorders, poor inhibitory control is also characteristic of substance-related disorders. Prolonged SSRT is found in chronic methamphetamine users^42^, cocaine users^43^, and alcohol-dependent users^44^. However, the present findings support the notion that impaired response inhibition is a general feature of OCD, in spite of the clinical heterogeneity that these patients present. Two neuroimaging studies have investigated the effects of OCD treatment on response inhibition^34^,^35^. Nakao *et al.* (2005) reported that pharmacotherapy or behavioral therapy in OCD patients increased task-relevant brain activation during performance of an interference control task in addition to improving symptoms^45^. However, due to the study design, it is not clear whether this change in activation is secondary to symptom improvement or instead due to the treatment. Another study found increased activation of multiple cortical and subcortical brain areas during a go/no go task in OCD patients treated with selective serotonin reuptake inhibitors (SSRIs) compared to OCD patients not treated with SSRIs^46^. However, this study was cross-sectional, had a small sample size, and did not identify the effects of the pharmacotherapy specifically on response inhibition. Additional results from neuropsychological studies have been inconsistent^47^,^48^. Thus it is not yet possible to conclude whether changes in response inhibition occur following treatment in OCD patients. Although our findings provide evidence that deficits in response inhibition are stable across symptom expression and severity, further investigation using a pre-post-treatment design will be necessary to explore whether response inhibition deficits are state dependent or trait-like.

There are several potential limitations to the present study. First, the recruited patients were not stratified for symptoms along the symptom dimensions; therefore, patients with high expression on specific dimensions may be underrepresented, and it was not possible to examine patients that exclusively expressed symptoms in one dimension, e.g., hoarding/symmetry. Second, the applied method for quantifying symptom Y-BOCS dimension scores neglected possible differences in symptom severity for the dimensions, as this measure quantifies only the presence or absence of a certain symptom. Thus, significant relations between overall symptom severity measures and response inhibition were not disclosed. Third, we showed the independence of response inhibition from symptom state using correlation and regression analysis with a cross-sectional approach. However, longitudinal data in patients with OCD comparing response inhibition before and after therapy are needed to strengthen these results.

In conclusion, the present study revealed that deficits in response inhibition are a common correlate of OCD that is independent of symptom severity and symptom dimensions. Thus, alterations in response inhibition represent a factor in the pathophysiology of OCD in general.

## Methods and materials

### Participants

Forty-two medication-free patients with OCD (19 females) and 42 healthy comparison participants (18 females) participated in the current study (see Table 1 for further characteristics). These patients were recruited from the psychology clinic at Second Xiangya Hospital of Central South University in China. All patients were diagnosed by trained clinicians using the Structured Clinical Interview for DSM-IV (SCID)^49^ and fulfilled criteria for OCD. We excluded subjects who met criteria for depression or any other comorbid current psychiatric disorders (Axis I or Axis II), and subjects who had a history of drug abuse, traumatic brain injury, or medical or neurological disorders, including tic and Tourette’s disorders. Healthy controls that were matched for age, gender, and years of education were recruited from the community and from Central South University. These control subjects reported no history of neurological or psychiatric disorders and were unmedicated. All subjects were self-reported right-handed, and had normal or corrected visual acuity and normal color vision. This study was conducted in accordance with the Declaration of Helsinki and was approved by the Ethics Committee of the Second Xiangya Hospital of Central South University. All subjects provided written informed consent according to institutional guidelines.

All participants completed the Beck Depression Inventory (BDI-II)^50^ and Obsessive-Compulsive Inventory-Revised (OCI-R)^51^. All patients were assessed for OCD symptom severity by a trained clinician using the Y-BOCS, including the checklist^10^.

### Symptom dimensions of OCD

Dimension scores of OCD symptoms were determined for each patient using the Y-BOCS-SC according to a method recently described by Katerberg *et al.*^3^. The five symptom dimensions are as follows: taboo, contamination/cleaning, doubt, rituals/superstitious, and hoarding/symmetry. Briefly, for the lifetime score, each item of the Y-BOCS-SC was coded with a score of 1 when the symptom was reported in the past or present, and with a score of 0 if the patient had never had the symptom. For present symptom scores, Y-BOCS-SC items were coded as 1 if the symptom was reported currently (past 1 week) and as 0 if the symptom was currently not experienced. Mean scale scores for each of the five symptom dimensions were computed for each patient by summing up item scores and dividing the sum by the total number of items of the respective dimension, resulting in a score ranging between 0 and 1 for present and lifetime symptoms, respectively.

### Task

The stop-signal task required subjects to perform a primary simple reaction time (go) task involving visual stimuli (the letter ‘X’). Go stimuli appeared as black upper-case letters (4 cm high × 4 cm wide) on a white background in the center of a 14 inch (in) computer screen placed approximately 60 cm in front of the subject at eye level. The thumb of the subject’s dominant hand was used to press the space key on a computer keyboard. On go trials (70%), a single go stimulus was presented. Each trial began with a black central fixation dot that was courier new typeface at point sizes of 34 for 500 ms, followed by the letter for that trial for 1000 ms, and then a blank screen for 1000 ms. On stop-signal trials (30%), the color of the ‘X’ changed from the black to red, signaling subjects to inhibit their response to the primary task. Subjects completed one practice block of 20 trials and four experimental blocks of 100 trials each. The stop-signal delay was 250 ms, and increased or decreased by 50 ms when subjects succeeded or failed to stop, respectively, in order to provide an approximately equal number of failed and successful stop-trials. The upper limit for stop-signal delay was 1000 ms, and the lower limit was 0 ms, i.e., concurrent with go stimulus onset.

Subjects were instructed to respond as quickly as possible to the go stimuli and to attempt to withhold that response if a stop signal occurred. The instruction to the participants emphasized response speed on go trials more than successful inhibition on stop trials. Subjects were told that they would be unable to inhibit their responses every time and that they should not wait for the stop signal. All subjects received the same instructions.

### Electrophysiological Recording and Measures

Electroencephalographic (EEG) activity was recorded using a 32-channel cap (Easy-cap), with a set of 30 Ag/AgC1 electrodes placed according to the 10/20 system. Electro-oculograms (EOG) were recorded via electrodes placed on the bilateral external canthi, and the left infraorbital and supraorbital areas to monitor for eye movements and blinks. Both EEG and EOG were sampled at 1000 Hz with a 0.1-200 Hz band pass using a Neuroscan NuAmps digital amplifier system (Neuroscan Inc., USA). The left mastoid was used as reference during recording, and an average of the right and left mastoid references was calculated off-line. Electrode impedances were kept below 5 kΩ.

Neuroscan analysis software (Scan 4.3, NeuroScan Inc., USA) was used to analyze EEG data offline. EOG artifacts were corrected using a correlation method. The EEG was then segmented in epochs of 100 ms pre-stimulus to 800 ms post-stimulus onset, and the pre-stimulus baseline was corrected. Segments contaminated with artifacts exceeding amplitude of ± 100 μV were excluded from averaging. Following this procedure, the averaged event-related potentials (ERPs) were low pass filtered at 30 Hz (24 dB/octave). ERPs were averaged to visual stop signals when responses were successfully inhibited (termed successful inhibition [SI]) and not inhibited (i.e., a button press on a stop-signal trial, termed failed inhibition [FI]).

Peak amplitudes and latencies were quantified within a predetermined latency window by means of an automatic peak-picking program. Peak amplitude and latency were measured relative to a 100 ms prestimulus baseline period, and latency was locked to the site of maximum amplitude. Components identified were N2 (180 to 350 ms) locked at electrode Fz, as well as P3 (250 to 400 ms) locked at electrode Cz.

### Statistical analyses

T-tests were used to compare the groups on demographics (age, years of education), as well as psychometric and task performance variables. For stop-signal trials, three-way repeated measures ANOVAs were used to analyze N2, P3 components with lead (Fz, Cz, Pz), and Trial Type (SI vs. FI) as within-subject factors, and Group (OCD patients, healthy controls) as a between-subjects factor. Since N2 and P3 were individually most pronounced at Fz and Cz, respectively, ERP amplitudes at these electrode sites were used for correlation and regression analyses. All statistical tests were two-tailed, using a significant level of α = 0.05. Post hoc tests with a Bonferroni adjustment for *p*-values were used. A sample size of 38 patients per group would be needed to detect a medium effect size (*f* = 0.25) between two groups with 80% power at 5% significance level. With adjusting the possible 10% attrition rate for EEG data offline analysis due to insufficient available numbers of correct artifact-free trials, the final sample size would be inflated into 84 in total. G-power (version 3.1.9.2) was used to perform power analysis.

Correlation analyses (Pearson *r*) were used to examine associations between indexes of response inhibition (behavior and ERPs indexes with significant group effects were chosen) and demographic variables in the whole sample, as well as clinical variables in patients. Multiple regression analyses, including the partial correlations, were performed for the OCD patient group by analyzing the prediction of response inhibition by the present and lifetime symptom dimension scores from the Y-BOCS-SC in order to explore the unique contributions of symptom dimensions.

## Author Contributions

X.Z.Z. designed research; H.L., J.F., J.J.D., C.Z, X.C.Z., and M.T.Z. performed research; H.L. analyzed data and wrote the paper. All authors reviewed the paper.

## Additional Information

**How to cite this article**: Lei, H. *et al*. Is impaired response inhibition independent of symptom dimensions in obsessive-compulsive disorder? Evidence from ERPs. *Sci. Rep.* **5**, 10413; doi: 10.1038/srep10413 (2015).

## Figures and Tables

**Table 1:** Demographic, clinical, and performance measures of patients with OCD and healthy comparison participants.

	**OCD patients (n = 42)**		**Healthy controls (n = 42)**		***p*****-value**
**Measures**	**Mean**	**SD**	**Mean**	**SD**	
**Demographic**					
Age (years)	21.67	4.76	22.79	3.57	ns
Education (years)	13.55	2.55	14.14	1.91	ns
Gender ratio (M/F)	23/19		24/18		ns

**Clinical**					
BDI-II	23.24	10.61	7.29	6.71	<.001
OCI-R	24.67	14.67	9.52	8.20	<.001
Y-BOCS	30.33	7.23	—	—	

**Task performance**					
Median Go RT (ms)	619.62	193.70	681.71	192.92	ns
Success rate in go trials (%)	0.89	0.10	0.88	0.07	ns
Successful inhibition (%)	59.87	8.91	60.74	7.19	ns
SSRT (ms)	212.62	51.04	174.51	66.77	<.01

**Table 2:** Scores and presence of current and lifetime symptom dimensions on the Y-BOCS Symptom checklist in 42 patients with OCD.

	**Present symptoms**		**Lifetime symptoms**	
	**Mean (SD)**	**Number of patients (%)**	**Mean (SD)**	**Number of patients (%)**
Taboo	0.12 (0.13)	23 (55)	0.16 (0.20)	24 (57)
Contamination/cleaning	0.13 (0.17)	25 (60)	0.16 (0.18)	29 (69)
Doubts	0.21 (0.20)	27 (64)	0.24 (0.21)	30 (71)
Superstitions/rituals	0.08 (0.12)	13 (31)	0.11 (0.15)	16 (38)
Symmetry/hoarding	0.30 (0.30)	28 (67)	0.33 (0.31)	29 (69)

**Table 3:** Multivariate regression model and correlation for the indexes of response inhibition as dependent variable and past and current symptom dimension scores as predictors.

	**SSRT**			**Amplitude of Stop-N2 in SI**			**Amplitude of Stop-N2 in FI**		
	***R***^***2***^	***β***	***r*****(partial)**	***R***^***2***^	***β***	***r*****(partial)**	***R***^***2***^	***β***	***r*****(partial)**
**Present symptoms**	0.07			0.12			0.09		
Taboo		0.27	0.17		−0.10	−0.06		−0.01	−0.01
Contamination/cleaning		0.10	0.07		0.36	0.26		0.29	0.21
Doubts		0.08	0.05		−0.29	−0.18		−0.22	−0.15
Superstitions/rituals		−0.13	−0.10		−0.02	−0.01		−0.06	−0.05
Symmetry/hoarding		−0.12	−0.01		0.01	0.00		−0.09	−0.06
**Lifetime symptoms**	0.07			0.10			0.10		
Taboo		0.28	0.15		−0.01	0.00		0.05	0.03
Contamination/cleaning		0.08	0.06		0.40	0.26		0.12	0.09
Doubts		0.10	0.07		−0.20	−0.13		−0.19	0.13
Superstitions/rituals		−0.19	−0.15		−0.13	−0.09		0.21	0.17
Symmetry/hoarding		−0.12	−0.08		−0.08	−0.06		−0.22	−0.15

*β* = standardized coefficient, SI = successful inhibition, FI = failed inhibition.
